# New fossil lizard specimens from a poorly-known squamate assemblage in the Upper Cretaceous (Campanian) San Juan Basin, New Mexico, USA

**DOI:** 10.7717/peerj.8846

**Published:** 2020-04-17

**Authors:** C. Henrik Woolley, Nathan D. Smith, Joseph J.W. Sertich

**Affiliations:** 1 Dinosaur Institute, Natural History Museum of Los Angeles County, Los Angeles, CA, United States of America; 2 Department of Earth Sciences, University of Southern California, Los Angeles, CA, United States of America; 3 Department of Earth Sciences, Denver Museum of Nature & Science, Denver, CO, United States of America

**Keywords:** Squamata, Laramidia, Biogeography, Biodiversity, Data revision

## Abstract

Recent collection efforts in the upper Campanian (∼76-73.5 Ma) Fruitland and Kirtland formations of northwestern New Mexico have significantly increased the taxonomic diversity of lizards in this historically poorly understood squamate assemblage. New lizard specimens from the “Hunter Wash Local Fauna” of the upper Fruitland and lower Kirtland formations include: (1) new specimens referable to Chamopsiidae; (2) new material belonging to Scincomorpha, (3) new material belonging to Anguidae; and (4) the first reported predatory lizard (Platynota) material from the Campanian of New Mexico. The increase in lizard diversity in the “Hunter Wash Local Fauna” expands our understanding of Late Cretaceous squamate taxonomy, distribution, and diversity in the Western Interior of North America (Laramidia). Collectively, the described specimens represent family-level diversity similar to that seen in other Campanian foreland basin deposits of the Western Interior, such as the mid-paleolatitude Kaiparowits Formation of southern Utah, the higher paleolatitude Dinosaur Park Formation of southern Alberta, and the lower paleolatitude Aguja Formation of southwestern Texas. The lizards of the “Hunter Wash Local Fauna” represent crucial mid-paleolatitude data from a coastal plain depositional setting in Laramidia—allowing for comparisons to more well-studied assemblages at different latitudes and in different depositional settings.

## Introduction

The Campanian Fruitland and Kirtland formations (∼76-73.5 Ma) (Fassett & Heizler, 2017) of northwestern New Mexico have a rich history of fossil macrovertebrate collection (Osborn & Sternberg, 1923; Ostrom, 1963; Carr & Williamson, 2010), but the corresponding microvertebrate assemblage remains understudied. In particular, squamate (lizards and snakes) descriptions have been limited to preliminary taxonomic studies (Armstrong-Ziegler, 1978; Armstrong-Ziegler, 1980; Sullivan, 1981). To date, the only formally described squamate taxa from the Fruitland and Kirtland formations are the chamopsiids *Chamops segnis* and *Leptochamops denticulatus* (Armstrong-Ziegler, 1978; Armstrong-Ziegler, 1980; Sullivan, 1981), the anguid c.f. *Gerrhonotus* sp. (Armstrong-Ziegler, 1980), and the ophidian *Coniophis cosgriffi* (Armstrong-Ziegler, 1978). All four of these taxonomic assignments, while important for establishing the presence of squamates in the Fruitland and Kirtland Formations, have been met with skepticism (Table 1; Estes, 1983; Gao & Fox, 1996; Nydam & Voci, 2007; Longrich, Bhullar & Gauthier, 2012a; Nydam, 2013b).

**Table 1:** Summary of previously described squamate material from the Fruitland and Kirtland formations, including suggested changes in taxonomic referral.

***Original Taxonomic Assignment (Armstrong-Ziegler, 1978; Armstrong-Ziegler, 1980; Sullivan, 1981)***	***Suggested Taxonomic Change***
**Scincomorpha**	**Lissamphibia** (Gao & Fox, 1996; Nydam & Voci, 2007) **Albanerpetontidae** (Present study)
Borioteiioidea	
Chamopsiidae	
*Leptochamops denticulatus*	
**Scincomorpha**	**Teiidae gen. et sp. indet.** (Gao & Fox, 1996); ***Chamops*sp.** (Nydam & Voci, 2007); **Chamopsiidae indet.** (Nydam, 2013b; present study)
Borioteiioidea	
Chamopsiidae	
*Chamops segnis*	
**Anguimorpha**	***Odaxosaurus*** (Gao & Fox, 1996; Nydam, 2013b; present study); **c.f.*Gerrhonotus*sp.** (Estes, 1983)
Anguidae	
c.f. *Gerrhonotus sp.*	
**Serpentes**	**“unwarranted” generic referral** (Longrich, Bhullar & Gauthier, 2012a; Longrich, Bhullar & Gauthier, 2012b); **Serpentes indet.** (present study)
Alethenophidia	
*Coniophis cosgriffi*	

Previous authors (Gao & Fox, 1996; Nydam & Voci, 2007) concluded that the material referred to *Leptochamops* by Armstrong-Ziegler (1978) and Armstrong-Ziegler (1980) can more confidently be referred to amphibian taxa. Indeed, the specimen figured in Armstrong-Ziegler (1980) is at least superficially most similar to amphibians belonging to the family Albanerpetontidae, which possess strongly pleurodont and non-pedicellate teeth bearing labiolingually compressed, usually tricuspid, chisel-like crowns (Sweetman & Gardner, 2012). We agree with previous authors (Gao & Fox, 1996; Nydam & Voci, 2007) that the material described and referenced in Armstrong-Ziegler (1978) and Armstrong-Ziegler (1980) (UALP 75137D, E, K, M, N) should at least be referred to Lissamphibia. Specimens belonging to Albanerpetontidae are common in recent collections of microvertebrate material from the Fruitland and Kirtland formations, and given the similarities between Albanerpetontid tooth-bearing elements and the described *Leptochamops* specimens in Armstrong-Ziegler (1978) and Armstrong-Ziegler (1980), we suggest that UALP 75137D, E, K, M, and N should be referred to Albanerpetontidae. Unfortunately, the material referred to *Leptochamops* in Armstrong-Ziegler (1978) and Armstrong-Ziegler (1980) cannot be located, and may have been lost in collections moves and loans between the University of Arizona Laboratory of Paleontology, Wayne State University, and the New Mexico Museum of Natural History & Science in the 1980s (E Lindsay, N Volden, pers. comm. to CHW, 2019).

Additionally, given the host of problematic referrals of Late Cretaceous squamate material to the species *Chamops segnis* (see Gao & Fox, 1996; Nydam, 2013a; Nydam, 2013b for extensive discussion), the referral of fragmentary material to *Chamops segnis* by Armstrong-Ziegler (1980) and Sullivan (1981) should also be regarded as extremely tentative. The specimen MNA Pl. 1613 in Armstrong-Ziegler (1980) is too fragmentary for a confident referral to *Chamops* given the more recently updated definitions of the genus by Gao & Fox (1996), Nydam & Voci (2007), and Nydam (2013b), who collectively conclude that a referral to Teiidae, gen. et. sp. indet. (Gao & Fox, 1996), or *Chamops sp*. (Nydam & Voci, 2007), or Chamopsiidae indet. (Nydam, 2013b), is more appropriate. The referral of UNM FKK-038a to *Chamops segnis* by Sullivan (1981) was also questioned by Gao & Fox (1996) and Nydam (2013b), with both studies concluding that, though referral of UNM FKK-038a to Teiidae is appropriate, referral to *Chamops segnis* is not justified. UNM FKK-038a may have been lost in the transfer of University of New Mexico specimens over to the New Mexico Museum of Natural History (N Volden, pers. comm. to CHW, 2019), and could not be re-evaluated in the current study.

As discussed by Nydam (2013b) and Gao & Fox (1996), the referral of fragmentary anguid material from the Fruitland/Kirtland formations to *Odaxosaurus* is more appropriate than the previous referral to c.f. *Gerrhonotus* sp. by Armstrong-Ziegler (1980) and Estes (1983). Unfortunately, the material referred to c.f. *Gerrhonotus* sp. in Armstrong-Ziegler (1980) seems to have also been lost in collections moves and loans between the University of Arizona Laboratory of Paleontology, Wayne State University, and the New Mexico Museum of Natural History & Science in the 1980s (E Lindsay, N Volden, pers. comm. to CHW, 2019). Additionally, the referral of fragmentary snake vertebrae to the ophidian genus *Coniophis* (Armstrong-Ziegler, 1978) was questioned in a recent review of the genus (Longrich, Bhullar & Gauthier, 2012a, Supp. 1). Although referral of MNA Pl. 1612 to *Coniophis* may not be appropriate, the specimen most certainly belongs to a snake, due to the presence of zygosphene/zygantrum articulations, with zygosphene articular surfaces that face ventrolaterally (Conrad, 2008: character 235, state 2) and a “haemal” keel on the ventral surface of the centrum (Longrich, Bhullar & Gauthier, 2012a; Xing et al., 2018). We therefore suggest that MNA Pl. 1612 be referred to as Serpentes indet. until more complete material is recovered.

In our review of historical data for the squamate fauna of the Fruitland and Kirtland formations, we encounter two major problems that have been previously summarized (Gao & Fox, 1996; Nydam, 2013b): **(1)** fragmentary data/incomplete specimens; and **(2)** outdated and/or inaccurate taxonomic referrals in previous research, which have both led to a lack of clarity in the true taxonomic composition of squamates during the Late Campanian in New Mexico. The study herein seeks to address these problems by describing newly collected squamate specimens from the Fruitland and Kirtland formations within the context of the previous work summarized above. This study includes descriptions of specimens referable to Chamopsiidae, Scincomorpha, Anguidae, and Platynota from the “Hunter Wash Local Fauna” in the Upper Fruitland and Lower Kirtland formations. The described specimens expand the taxonomic and morphological diversity of lizards within the “Hunter Wash Local Fauna” and represent significant new data points in understanding the regional distribution of lizard groups during the Campanian in western North America.

Discussions of biogeographic patterns in Late Cretaceous North American lizards (Nydam, 2013b; Nydam, Rowe & Cifelli, 2013) have cautiously incorporated the previous erroneous identifications of squamates from the Fruitland & Kirtland formations (Armstrong-Ziegler, 1978; Armstrong-Ziegler, 1980; Sullivan, 1981). At a family taxonomic level, the updated lizard assemblage of the Fruitland and Kirtland formations—representing an important mid-paleolatitude coastal plain depositional setting (Gates et al., 2010)—allow for comparison to well-sampled lizard assemblages from: **(1)** coastal plain depositional settings at higher paleolatitudes (Dinosaur Park Formation—Gao & Fox, 1996), and lower paleolatitudes (Aguja Formation—Nydam, Rowe & Cifelli, 2013; Cerro del Pueblo Formation—Aguillon-Martinez, 2010), and **(2)** alluvial plain depositional settings at mid-paleolatitudes (Kaiparowits Formation—Nydam, 2013a) and at higher paleolatitudes (Wapiti Formation—Nydam, Caldwell & Fanti, 2010; Nydam, 2013b), (Table 2). The specimens described in detail herein ensure that accurate data from the squamate assemblage of the Fruitland and Kirtland formations can be used for reconstructions of local and regional biogeographic patterns in the future.

### Geologic Setting

**Figure 1:**
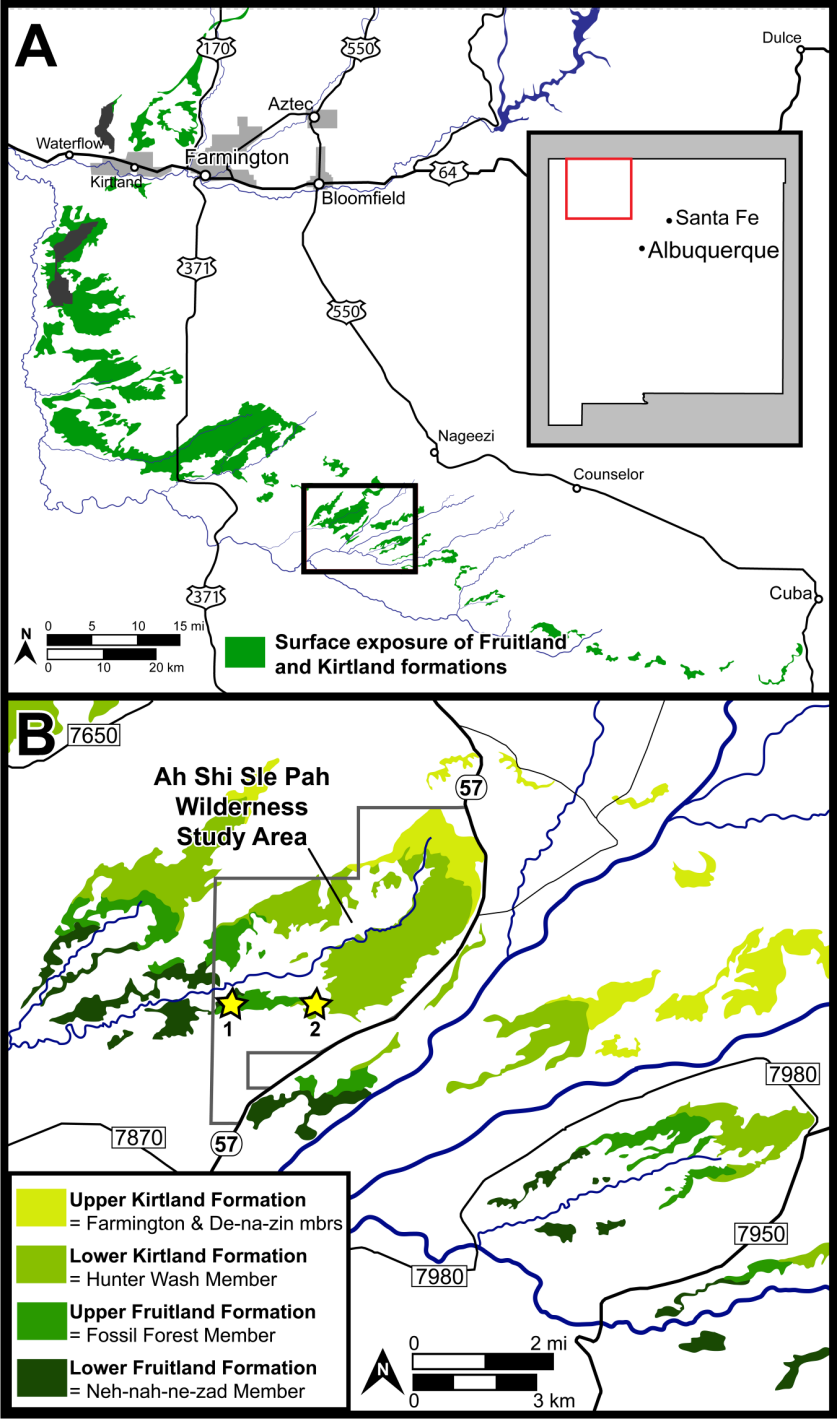
Surface exposures of the Fruitland and Kirtland formations in the San Juan Basin of northwestern New Mexico (A), with a detailed map of the region surrounding the Ah-Shi-Sle-Pah Wilderness Study Area (B). Approximate locations of the two localities discussed are indicated with stars: 1 = DMNH loc. 6685 “Black Bowl”, 2 = DMNH loc. 5204 “Tom’s Dirty Hole”. Map modified from Joyce, Lyson & Sertich (2018).

DMNH loc. 6685, “Black Bowl”, is located in a gray fine-grained sandstone with carbon staining within the upper Fruitland Formation (Fig. 1). DMNH loc. 5204, “Tom’s Dirty Hole”, (Fig. 1) is located in a gray fine-grained sandstone within the lower Kirtland Formation. Though exact stratigraphic location is difficult to discern based on variable interpretations of the interformational contact, both localities lie well within the “Hunter Wash Local Fauna” defined as the fossiliferous horizons in the upper 12.2 m of the Fruitland Formation and the lower 16.8 m of the Kirtland Formation in the Hunter Wash area (Clemens, 1973; Sullivan & Lucas, 2003; Sullivan & Lucas, 2006). Joyce, Lyson & Sertich (2018) called attention to the fact that the definition of the “Hunter Wash Local Fauna” is dependent on a clear interformational contact between the Fruitland and Kirtland formations. This is problematic given that considerable disagreement persists over the nature and stratigraphic location of the contact between the Fruitland and Kirtland formations. The Fruitland/Kirtland contact was initially recognized as gradational, though the underlying Fruitland Formation was observed to be typically sandier than the overlying Kirtland Formation (Bauer, 1916). Later work (Fassett & Hinds, 1971; Fassett, 2000; Fassett, 2010) defined the contact as the top of the highest coal or carbonaceous shale in the Fruitland Formation. Given the gradational transition between the Upper Fruitland Formation and the lower shale member of the Kirtland Formation (‘Hunter Wash Member’ of Hunt & Lucas, 1992), the top of the highest coal/carbonaceous shale is often obscured in outcrop. Other attempts to define the Fruitland/Kirtland contact (Hunt & Lucas, 1992; Lucas, Hunt & Sullivan, 2006) have placed the top of the Fruitland at the base of the ‘Bisti Member’ or ‘Bisti Bed,’ an indurated but laterally discontinuous sandstone horizon. More work is necessary before a robust lithostratigraphic definition of the Fruitland/Kirtland contact is accepted. Regardless of definition, both localities discussed herein, “Black Bowl” and “Tom’s Dirty Hole”, are well within the recognized horizons of the “Hunter Wash Local Fauna.”

## Materials & Methods

All materials herein were collected by Denver Museum of Nature & Science field crews during the 2014 and 2016 field seasons under United States Federal Bureau of Land Management (BLM) permit NM14-04S. Specimens were bulk collected in matrix using standard shovels and pick-axes, placed into 15-gallon plastic bags and transported to the Denver Museum of Nature & Science for wet screen-washing and sorting via the methods outlined in Cifelli, Madsen & Larson (1996), and identification. Specimens were measured and photographed at the Natural History Museum of Los Angeles County using a Keyence VHX-5000 digital microscope. Scanning electron micrographs of the specimens were produced using a Hitachi S-3000 N scanning electron microscope at the Natural History Museum of Los Angeles County. All specimens described in this study are housed in the Earth Sciences Vertebrate Paleontology collections at the Denver Museum of Nature & Science.

**Anatomical terminology:** We follow the recommendations of Richter (1994), Evans & Searle (2002), and Smith & Dodson (2003), regarding dental anatomical terminology.

## Results

### Systematic paleontology

**Table:** 

Reptilia Linnaeus, 1758
Squamata Oppel, 1811
Scincomorpha Camp, 1923
Boreoteiioidea Nydam, Eaton & Sankey, 2007
Chamopsiidae Denton & O’Neill, 1995

**Referred Specimen**: DMNH EPV.119583 (jaw fragment).

**Locality and Horizon:** DMNH loc. 6685 “Black Bowl”, Upper Fruitland Formation (Campanian), San Juan County, New Mexico. Within the Ah-She-Sle-Pah Wilderness Study Area.

**Table 2:** Squamate faunas of select Late Campanian geologic units in the Western Interior of North America. Geologic units arranged in descending order of geographic location, from North to South.

**Formation**	**Identified Scincomorpha**	**Identified Anguimorpha**	**Identified Serpentes**
**Wapiti** (Nydam, Caldwell & Fanti, 2010)—Central Alberta	**Scincomorpha**	None reported	None reported
	Borioteiioidea		
	Chamopsiidae		
	- *Socognathus unicuspis*		
	Scincomorpha indet.		
	- *Kleskunsaurus grandeprairiensis*		
**Dinosaur Park** (Listed as Oldman Formation in (Gao & Fox, 1996)—Southern Alberta	**Scincomorpha**	**Anguimorpha**	None reported
	Borioteiioidea	Xenosauridae	
	Chamopsiidae	-*?Exostinus sp.*	
	- *Chamops sp.*	Anguidae	
	*-Leptochamops*	-*Odaxosaurus priscus*	
	*-Socognathus*	Platynota	
	Scincidae	-*Parasaniwa* n. sp.	
	*-Orthrioscincus*	-c.f. *P. wyomingensis*	
	?Cordylidae	Helodermatidae	
	Incertae sedis	-*Labriodectes*	
	*-Glyptogenys*	Varanidae	
	*-Sphenosiagon*	-*Palaeosaniwa*	
	*-Gerontoseps*		
**Kaiparowits** (Nydam, Eaton & Sankey, 2007; Nydam & Voci, 2007; Nydam, 2013a)—Southern Utah	**Scincomorpha**	**Anguimorpha**	**Serpentes**
	Borioteiioidea	Anguidae	Alethenophidia
	-*Peneteius saueri*	-*Odaxosaurus roosevelti*	-*Coniophis* sp.
	Chamopsiidae	-*Odaxosaurus priscus*	
	*-Meniscognathus molybrochoros*		
	-*Chamops sp. c.f. C. segnis*	Xenosauridae	
	-*Leptochamops*	-*?Exostinus*	
	-*Tripennaculus eatoni*		
	Contogeniidae	Platynota	
	-*Palaeoscincosaurus pharkidodon*	-*Parasaniwa cynochoros*	
	c.f. Scincoidea	Kaiparowits Morphotype H-J	
	Paramacellodid/Cordylid Grade		
	Kaiparowits Morphotype A-G		
**Fruitland/Kirtland***(Updated with current taxonomic IDs)*—Northwest New Mexico	**Scincomorpha**	**Anguimorpha**	**Serpentes**
	Borioteiioidea	Anguidae.	-Serpentes indet.
	-Chamopsiidae indet.	-*Odaxosaurus* sp.	
		-Anguidae indet.	
	Scincomorpha indet.		
	-?Scincidae	Platynota	
		-Platynota indet.	
**Aguja** (Nydam, Rowe & Cifelli, 2013)—Southwestern Texas	**Scincomorpha**	**Anguimorpha**	**Serpentes**
	cf. Xantusiidae	Anguidae	-Serpentes indet.
	-*Catactegenys solaster*	-*Odaxosaurus piger*	
		-Anguidae indet.	
	Scincomorpha indet.	-aff. Xenosauridae	
	-*Apsgnathus triptodon*		
	-Unnamed scincid-grade taxon	Platynota	
	-aff. Scincomorpha	-cf. *Parasaniwa wyomingensis*	
**Cerro del Pueblo** (Aguillon-Martinez, 2010)—Coahuila, Mexico	**Scincomorpha**	**Anguimorpha**	**Serpentes**
	Borioteiioidea	Anguidae	Alethenophidia
	Chamopsiidae	-*Odaxosaurus* new sp.	- *Coniophis* sp.
	-*Peneteius* sp.	Platynota	
		-cf. *Parasaniwa wyomingensis*	
		Helodermatidae	
		-*Paraderma* cf. *P. bogerti*	
		Varanidae	
		-*Palaeosaniwa* c.f. *P. canadensis*	

**Figure 2:**
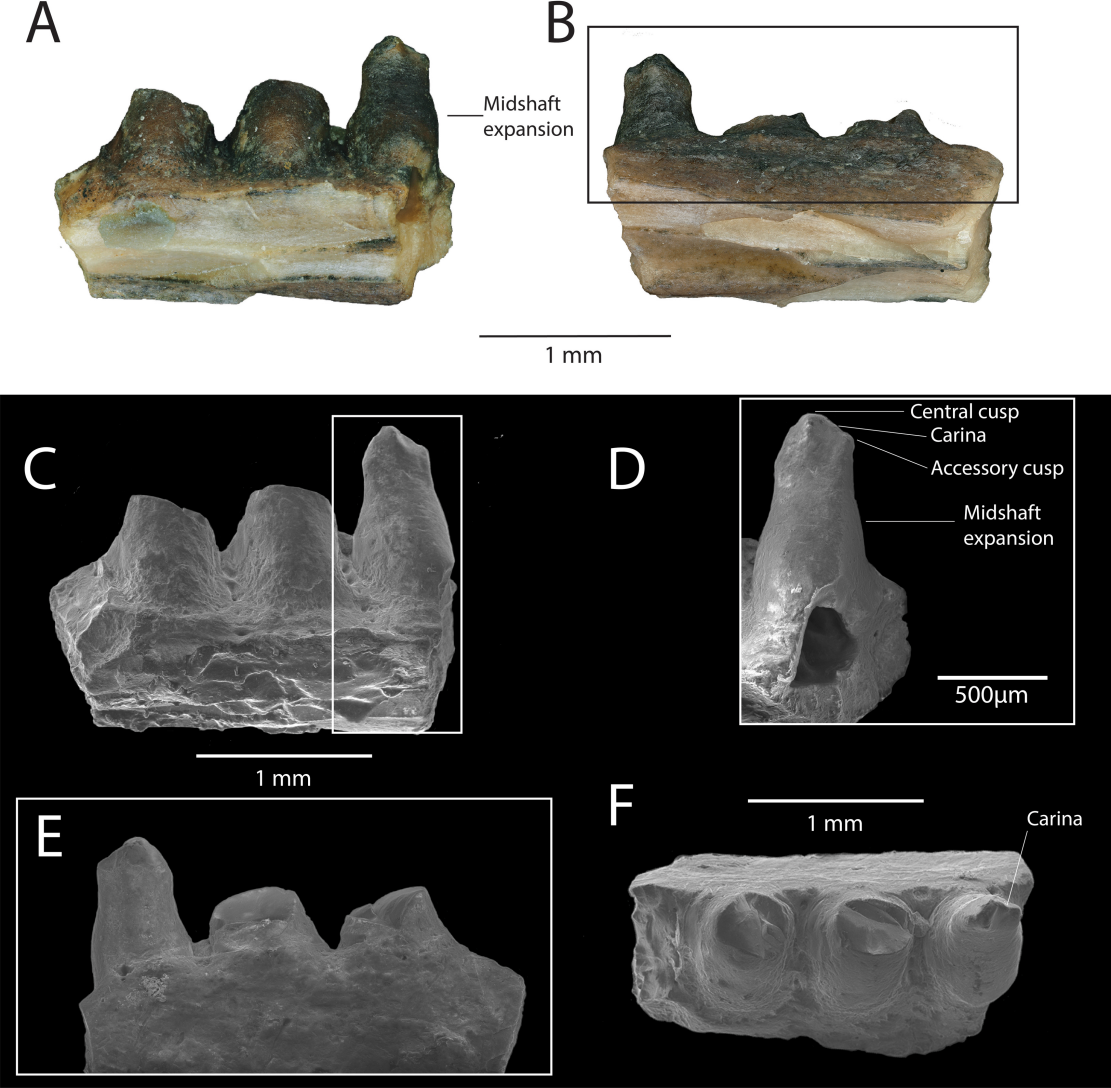
DMNH EPV.119583, Chamopsiidae indet. jaw fragment from the “Hunter Wash Local Fauna”, Fruitland Formation. (A) Medial view. (B) Lateral view. (C) Scanning electron micrograph, medial view. (D) Scanning electron micrograph, medial view, perpendicular to labiolingual axis of complete tooth crown. (E) Scanning electron micrograph, lateral view. (F) Scanning electron micrograph, occlusal view.

**Description:** DMNH EPV.119583 (Figs. 2A–2F) is a jaw fragment with three partial teeth preserved. The bone to which the teeth attach is too incomplete to assess whether this specimen belongs to the maxilla or the dentary, but the preserved teeth are informative enough to merit description. Tooth attachment is subpleurodont, with the lateral parapet extending less than one third the length of the tooth shaft. Teeth are subcircular in cross-section, and are widely spaced along the tooth row. The bases of the teeth are labiolingually expanded at their mid-shaft, and taper in width occlusally, giving the tooth a barrel-like shape (Figs. 2C–2D). One tooth (Fig. 2D) is almost completely preserved, with the tooth crown partially chipped off. This tooth is straight throughout its long axis and not recurved. The crown preserves the base of a central cusp and a mesial/distal cusp (orientation cannot be determined) that are connected by a prominent carina (Figs. 2E–2F).

**Discussion**: The tooth attachment of DMNH EPV.119583 (subpleurodont), the tooth shaft shape (barrel-like), and the cross-sectional shape of the teeth (subcircular), are similar to that of Late Cretaceous chamopsiids in North America, particularly specimens associated with the genera *Chamops* (Maastrichtian Lance Formation, Wyoming, Marsh, 1892; Gilmore, 1928; Estes, 1964; Late Campanian Dinosaur Park Formation, Alberta, Maastrichtian Frenchman Formation, Saskatchewan, Gao & Fox, 1996; Late Campanian Kaiparowits Formation, Nydam & Voci, 2007, Turonian Smoky Hollow Member of the Straight Cliffs Formation, Utah, Nydam, 2013a), *Leptochamops* (Maastrichtian Lance Formation, Wyoming, Gilmore, 1928; Estes, 1964; Late Campanian Dinosaur Park Formation, Maastrichtian Frenchman Formation, Alberta Gao & Fox, 1996; Late Campanian Kaiparowits Formation, Utah, Nydam & Voci, 2007), *Socognathus* (Late Campanian Dinosaur Park Formation, Alberta, Gao & Fox, 1996; Maastrichtian Lance Formation, Wyoming, Longrich, Bhullar & Gauthier, 2012b, and an unnamed chamopsiid from the Maastrichtian Laramie Formation, Colorado Longrich, Bhullar & Gauthier, 2012b). Subpleurodont teeth have been interpreted as a synapomorphy of Borioteiioidea (Nydam, Eaton & Sankey, 2007). Both barrel-shaped tooth cross sections and widely spaced tooth positions are considered diagnostic features of Chamopsiidae (Nydam, Caldwell & Fanti, 2010), supporting the referral of DMNH EPV.119583 to this family. Furthermore, the accessory cusp on the most complete tooth of DMNH EPV.119583, connected to the main cusp by a prominent carina, is also a diagnostic feature of Chamopsiidae (Nydam, Caldwell & Fanti, 2010). The most complete tooth in DMNH EPV.119583 is straight throughout its long axis, which distinguishes it from *Socognathus* and *Meniscognathus*, though some variation in this condition may be present in these taxa (Nydam, Caldwell & Fanti, 2010). The development of the prominent mesial/distal carina into an accessory cusp distinguishes DMNH EPV.119583 from other chamopsiid taxa such as *Pelsochamops* (Makádi, 2013) and *Socognathus* (Gao & Fox, 1991; Gao & Fox, 1996), in which the carina is not developed such that it forms a cusp. The fragmentary preservation of DMNH EPV.119583 limits our ability to make further taxonomic distinctions, but based on apomorphic features alone, the specimen at least belongs to a chamopsiid that has prominent mesial and distal carina that develop into accessory cusps.

### Systematic paleontology

**Table:** 

Scincomorpha Camp, 1923
Gen. et sp. indet.

**Referred specimen**: DMNH EPV.119554 (partial left dentary).

**Locality and horizon**: DMNH 5204, “Tom’s Dirty Hole”, Lower Kirtland Formation (Campanian), San Juan County, New Mexico. Within the Ah-She-Sle-Pah Wilderness Study Area.

**Figure 3:**
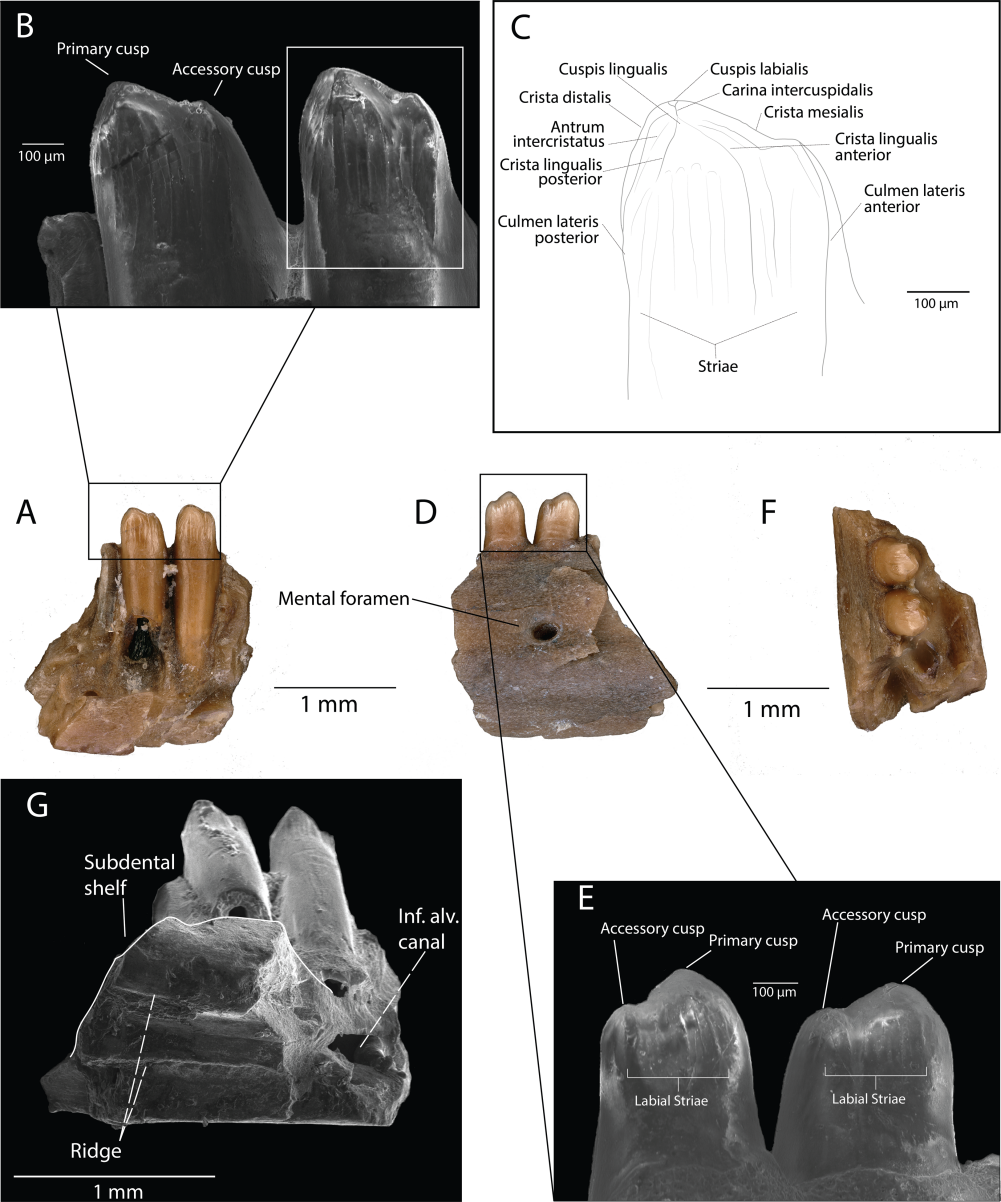
DMNH EPV.119554, Scincomorpha, partial left dentary, from the “Hunter Wash Local Fauna”, Kirtland Formation. (A) Medial view. (B) Medial view, zoom (scanning electron micrograph). (C) Line drawing of anterior tooth in medial view. (D) Lateral view. (E) Lateral view, zoom (scanning electron micrograph). (F) Occlusal view. (G) Ventromedial view (scanning electron micrograph). Abbreviations: Inf. Alv. Canal, Inferior Alveolar Canal.

**Description**: DMNH EPV.119554 (Figs. 3A–3G) is a partial left dentary with three tooth positions, two partial teeth and one complete tooth preserved. The medial and lateral walls of the Meckelian canal are not preserved, but the portion ventral to the subdental shelf remains and has two distinct parallel anteroposteriorly-directed ridges (Fig. 3G). The inferior alveolar canal has one branching canal that terminates laterally with a mental foramen (Fig. 3D). The subdental shelf is prominent and extends medially by twice the tooth shaft cross-sectional diameter. The medial margin of the subdental shelf thickens such that the dorsal surface of the subdental shelf curves dorsally (Fig. 3F). Tooth attachment is pleurodont, with the tooth attached to the lateral parapet for roughly two-thirds of its length (Fig. 3A). A nutrient foramen is present at the base of the posterior-most tooth position. Tooth shafts are slender and cylindrical. Tooth crowns are curved lingually. The tooth crowns are incipiently bicuspid, with the larger, primary cusp forming a medial apex and the smaller, accessory cusp developed on the mesial carina (Fig. 3B). The posterior cusp has a distinct cuspis labialis and a distinct cuspis lingualis, with a weakly-defined carina intercuspidalis connecting them (Fig. 3C). The cuspis labialis, which forms the apex of the cristae mesialis and distalis, is distinctly higher than the cuspis lingualis (the apex of the cristae lingualis anterior and posterior) (Fig. 3C). A weakly-defined posterior portion of the antrum intercristatus is visible on the posterior tooth crown (Fig. 3C). The culmen lateris anterior separates most of the anterior portion of the anterior cusp and wraps medially around the tooth shaft at a shallow angle. Vertical apical striae are present, with a larger vertical groove at the convergent margin between the two cusps. The lingual striae of the tooth crown terminate at the same height as the lateral parapet. Weak apical striae are present on the labial surface of the tooth crown.

**Discussion:** We interpret the structures converging at the cuspis lingualis in DMNH EPV.119554 as cristae lingualis (seen in scincids/lacertids, Kosma, 2004), rather than striae dominans (seen in paramacellodids, Kosma, 2004), because of several tooth crown features that are observed in scincids and lacertids and not paramacellodids, which are outlined below. **(1)** bicuspid teeth with a main cusp and a smaller mesial cusp set off from the main cusp can be observed in modern lacertine genera *Acanthodactylus*, *Algyroides*, *Archaeolacerta*, and *Darevskia* (Kosma, 2004). **(2)** *Alggyroides*, *Archaeolacerta*, and *Darevskia* also possess lingual apical striae similar to those seen in DMNH EPV.119554 (Kosma, 2004). **(3)** DMNH EPV.119554 also demonstrates tooth morphology similar to that of the fossil scincids *Orthrioscincus mixtus* from the Upper Campanian Dinosaur Park Formation (Gao & Fox, 1996), southeastern Alberta, Canada, and *Estescincosaurus cooki* (Estes, 1964; Sullivan, 1997) from the Maastrichtian Lance Creek Formation, Wyoming, USA, in possessing bicuspid teeth with a main cusp and a smaller mesial cusp set off from the main cusp, and a main cusp with lingual striae and weak labial striae. Paramacellodids, such as *Paramacellodus* and *Becklesius hoffstetteri* (Kosma, 2004), often possess chisel-shaped, unicuspid tooth crowns that are not similar to the morphology seen in DMNH EPV.119554. The cylindrical generalized scincomorphan tooth plan, and similarities with lacertid and scincid tooth crown morphologies, allow us to confidently place DMNH EPV.119554 within Scincomorpha. The temporal distribution of lacertids (only one tentative occurrence from the Mesozoic, (Gasca, Canudo & Moreno-Azanza, 2007) and the presence of scincids and lizards with scincid-grade tooth crown morphology in numerous geologic units in the Cretaceous of North America (Gao & Fox, 1996; Nydam, Rowe & Cifelli, 2013) suggest that DMNH EPV.119554 most likely belongs to a scincid. However, based solely on the morphological features of the specimen, we limit assignment of DMNH EPV.119554 to Scincomorpha until more complete material is recovered.

Scincomorpha family indeterminate

**Referred Specimen:** DMNH EPV.119567 (osteoderm)

**Locality and Horizon:** DMNH loc. 5204, “Tom’s Dirty Hole”, Lower Kirtland Formation (Campanian), San Juan County, New Mexico. Within the Ah-She-Sle-Pah Wilderness Study Area.

**Figure 4:**
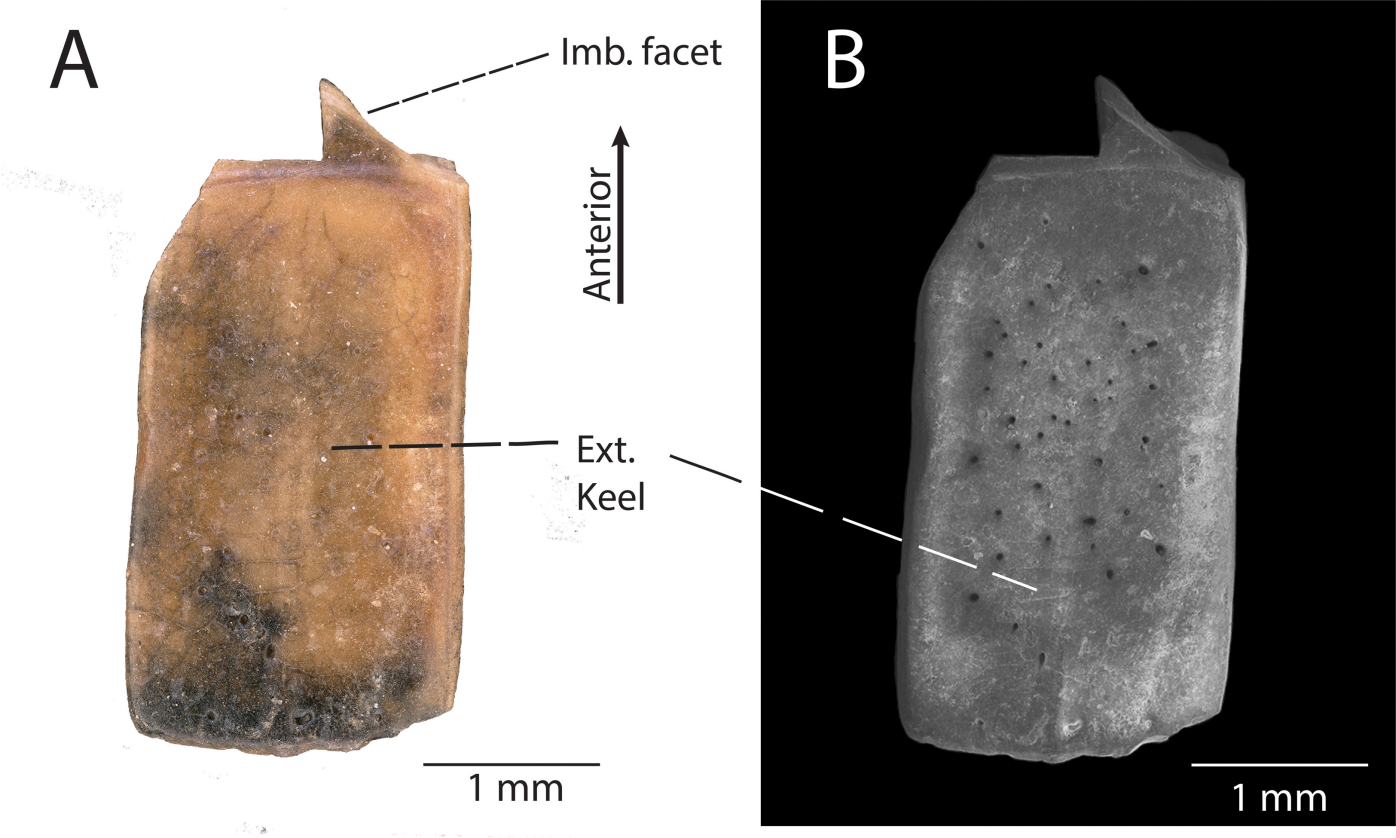
DMNH EPV.119567, Scincomorpha indet. osteoderm from the “Hunter Wash Local Fauna”, Kirtland Formation. (A) external view. (B) Scanning electron micrograph, external view. Abbrevations: Imb. facet, Anterior imbrication facet; Ext. keel, external keel.

**Description:** DMNH EPV.119567 (Fig. 4) is a rectangular osteoderm with a weak keel running longitudinally across the center of the external surface. The external surface of the osteoderm is generally smooth with small pits concentrated near the central keel (Fig. 4B). The external pits do not penetrate through to the internal surface of the osteoderm. The internal surface of the osteoderm is smooth and concave toward the external surface. The anterior imbrication facet is broken off, with only a small, triangular portion preserved (Fig. 4B).

**Discussion:** The presence of a central keel on the external surface of the osteoderm, the limited surficial sculpturing, and the large size of the osteoderm indicate that DMNH EPV.119567 belongs to a scincomorphan lizard (sensu Broschinski & Sigogneau-Russell, 1996). However, we cannot confidently associate this isolated osteoderm with any of the taxa described herein, nor can we confidently associate its anatomical position on the body. Similar large, rectangular osteoderms with a central external keel and limited surficial sculpturing have been assigned to Scincomorpha in both the paracontemporaneous Kaiparowits Formation southwestern Utah (Nydam, 2013a), and the paracontemporaneous Aguja Formation of southwestern Texas (Nydam, Rowe & Cifelli, 2013). Because these lizard assemblages bracket the Fruitland/Kirtland formations to the northwest (Kaiparowits Formation) and southeast (Aguja Formation), it is predictable to find scincomorphan-grade osteoderms within the “Hunter Wash Local Fauna”.

### Systematic paleontology

**Table:** 

Anguimorpha Fürbringer, 1900
Anguidae Gray, 1825
*Odaxosaurus*Gilmore, 1928
Species indet.

**Referred Specimen**: DMNH EPV.119555 (posterior left dentary).

**Locality and Horizon**: DMNH loc. 5204, “Tom’s Dirty Hole”, Lower Kirtland Formation (Campanian), San Juan County, New Mexico. Within the Ah-She-Sle-Pah Wilderness Study Area.

**Figure 5:**
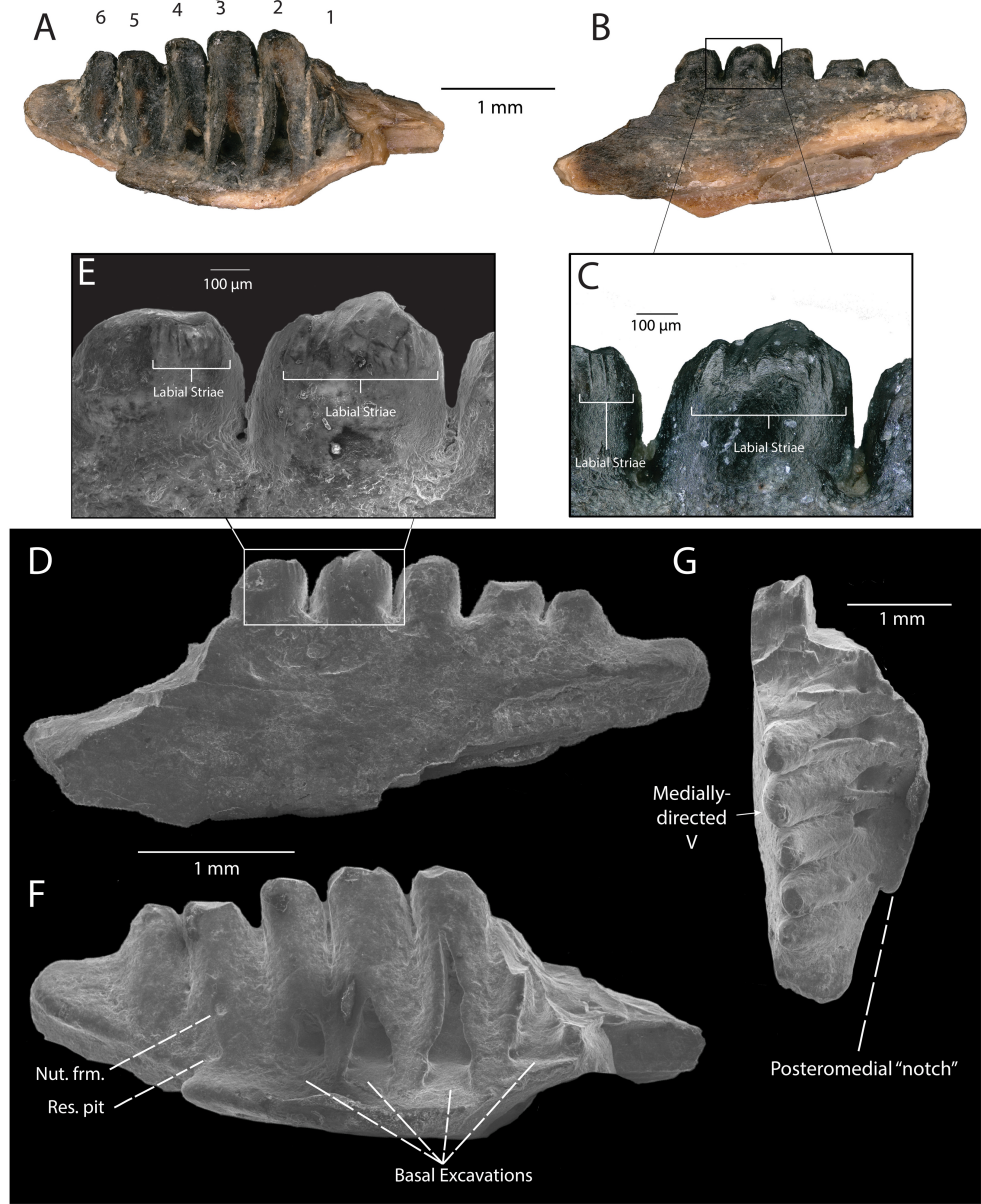
DMNH EPV.119555, Anguidae posterior left dentary from the “Hunter Wash Local Fauna”, Kirtland Formation. (A) Medial view. Numbers indicate preserved tooth positions: 1, anteriormost; 6, posteriormost. (B) Lateral view. (C) Lateral view, zoom. (D) Scanning electron micrograph, lateral view. (E) Scanning electron micrograph, lateral view, zoom. (F) Scanning electron micrograph, medial view. (G) Scanning electron micrograph, occlusal view. Abbreviations: Nut. frm., Nutrient foramen; Res. pit, Resorption pit.

**Description**: DMNH EPV.119555 (Figs. 5A–5G) is the posterior portion of a left dentary, indicated by the presence of a posteromedial “notch” in the subdental lamina (Fig. 5G). Six tooth positions are preserved (Fig. 5A), with the anterior-most tooth partially preserved (#1, Fig. 5A), and the five posterior-most teeth completely preserved (#2–6, Fig. 5A). Tooth attachment is pleurodont, with tooth bases labiolingually expanded and attached at an oblique angle to the subdental shelf (Fig. 5F). A faintly preserved nutrient foramina can be seen at the base of the second posteriormost tooth (Figs. 5F and 5G). Tooth shaft height and tooth base expansion lessens sharply from anterior to posterior. Tooth shafts are thick and stocky in overall shape, indicative of a more posterior position in the dentary. The four anteriormost teeth showcase triangular basal excavations posterolingually to the tooth bases (Figs. 5F and 5G), whereas the fifth anterior-most tooth showcases a circular resorption pit (Fig. 5F). This pattern is indicative of a progressive anterior-to-posterior tooth replacement strategy. Tooth crowns are incompletely preserved and do not preserve enamel on the lingual surface, but are gently recurved and chisel-shaped (Figs. 5B and 5F). The enamel on the third anteriormost tooth crown is preserved on the labial surface, and possesses weak labial apical striae (Figs. 5C and 5E). The cutting edge of the tooth crowns form a medially-pointed V-shape (Fig. 5G).

**Discussion**: DMNH EPV.119555 is referable to Anguimorpha based on synapomorphic features concerning the development of replacement teeth: **(1)** replacement teeth develop posterolingually to tooth midline (Estes, De Queiroz & Gauthier, 1988); **(2)** small resorption pits present (Estes, De Queiroz & Gauthier, 1988). Furthermore, DMNH EPV.119555 is referable to Anguidae on the basis of tooth crown morphology: **(1)** in occlusal view, cutting edge of posterior teeth forming an inwardly-pointing V (Fig. 5G); **(2)** crown apex lies slightly posterior and lingual to center of long axis of tooth (Figs. 5F and 5G); **(3)** crown is commonly rotated about the long axis of the tooth and apex tipped posteriorly, so that in profile the leading edge is prominently convex and extends back to the apex, giving a recurved, chisel-shape to the crown (Fig. 5F) (Estes, 1964). Additionally, striations on the labial surface of the tooth crown of DMNH EPV.119555 are noteworthy (Figs. 5C and 5E), since striations on both labial and lingual surfaces on the tooth crowns have historically been considered a diagnostic character of Anguidae (Gauthier, 1982; but recent evidence (Nydam, 2013a) suggests that lingual striations on tooth crowns may not be a requisite diagnostic character in anguid taxa such as *Odaxosaurus roosevelti*). The lack of preservation of lingual surfaces of the teeth of DMNH EPV.119555 means we cannot assess this character fully. However, because striae are present at least on the labial surface of the tooth crown of DMNH EPV.119555, this specimen can be used to make further taxonomic distinctions pending the recovery of more complete material.

The only known anguid taxa from the Late Campanian of North America belong to the genus *Odaxosaurus*: *O. piger* from the Aguja Formation (Nydam, Rowe & Cifelli, 2013), *O. priscus* and *O. roosevelti* from the Kaiparowits Formation (Nydam, 2013a), *O. priscus* from the Dinosaur Park Formation (Gao & Fox, 1996), and *O.* sp. nov. from the Cerro del Pueblo Formation (Aguillon-Martinez, 2010). The close spacing of the teeth in DMNH EPV.119555 is similar to the close spacing of teeth in *O. roosevelti* (Nydam, 2013a) and *O. piger* (Nydam, Rowe & Cifelli, 2013), while the teeth in *O. priscus* (Nydam, 2013a) and *O.* sp. nov. (Aguillon-Martinez, 2010) are generally spaced further apart from one another. Additionally, the tooth crowns in DMNH EPV.119555 exhibit less “mesiodistal flaring” than that observed in *O. piger* (Nydam, 2013a; Nydam, Rowe & Cifelli, 2013) and *O.* sp. nov. (Aguillon-Martinez, 2010), but this could likely to be an artifact of poor preservation and/or tooth enamel wear in DMNH EPV.119555. The preserved anatomical features highlighted above in DMNH EPV.199555, in addition to the presence of three species of *Odaxosaurus* in geographically bracketing paracontemporaneous lizard faunas (Kaiparowits Formation, southern Utah; Aguja Formation, southwestern Texas), merit a tentative referral to *Odaxosaurus* sp. pending the recovery of more complete material. Because the only other described specimens from the Fruitland/Kirtland formations (UALP 75137-F: left dentary fragment; UALP 75317-G: left dentary fragment; Armstrong-Ziegler, 1980) referable to *Odaxosaurus* cannot be located in museum collections, DMNH EPV.119555 is a critical representative specimen for the genus in the Late Campanian of New Mexico.

Anguidae indet.

“Hunter Wash” Anguidae Osteoderm Morphotype A

**Referred specimens:** DMNH EPV.119455 (trunk osteoderm), DMNH EPV.199457 (cranial osteoderm).

**Locality/Horizon:** DMNH loc. 6685 “Black Bowl”, Upper Fruitland Formation (Campanian), San Juan County, New Mexico. Within the Ah-She-Sle-Pah Wilderness Study Area.

**Figure 6:**
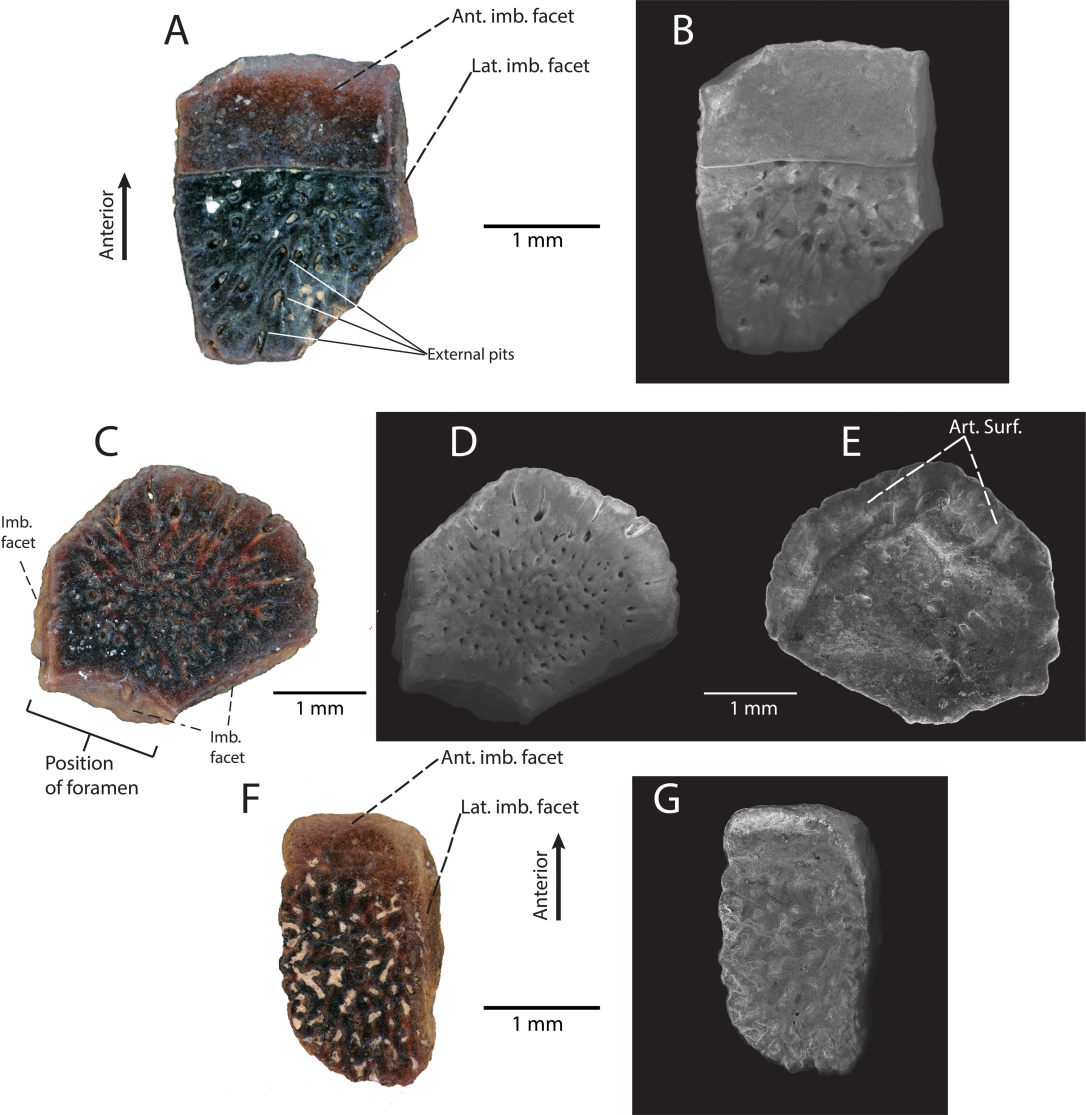
Anguidae indet. osteoderms from the “Hunter Wash Local Fauna”, Fruitland/Kirtland formations. (A–E) “Hunter Wash” Anguidae Osteoderm Morphotype A. (A) DMNH EPV.119455 in external view. (B) Scanning electron micrograph, external view. (C) DMNH EPV.119457 in external view. (D) Scanning electron micrograph, external view. (E) Scanning electron micrograph, internal view. (F–G) “Hunter Wash” Anguidae Osteoderm Morphotype B. (F) DMNH EPV.119577 in external view. (G) Scanning electron micrograph, external view. Abbreviations: Ant. imb. facet, Anterior imbrication facet; Art. Surf, Articular surface. Imb. facet, Imbrication facet; Lat. Imb. facet, Lateral imbrication facet.

**Description:** DMNH EPV.119455 (Figs. 6A and 6B) is a flat (i.e., no externally-directed curvature or keel) rectangular trunk osteoderm with a large, distinctive anterior imbrication facet and narrow lateral imbrication facets. The external surface is ornamented with a series of subcircular pits that are oriented such that their openings radiate laterally and away from the center of the osteoderm (Fig. 6A). The right lateral and posterior edge of the osteoderm is broken. DMNH EPV.119457 (Figs. 6C–6E) is a flat, hexagonal, cranial osteoderm with narrow imbrication facets on the anterior three sides of the specimen (Fig. 6C). The central imbrication facet is concave inward toward the center of the osteoderm (Figs. 6C–6D). This indicates that the osteoderm was located at the margin of a foramen in the skull, though because this specimen is isolated, it is difficult to determine its exact location. On the internal surface (Fig. 6E), two well-defined articular facets are angled toward the outer margin of the osteoderm for articulation with at least two additional osteoderms on the skull. The external surface of the osteoderm is heavily ornamented with a series of subcircular pits that are connected by vascular canals that radiate from the center of the osteoderm.

**Discussion:** *See below*.

“Hunter Wash” Anguidae Osteoderm Morphotype B

**Referred Specimens:** DMNH EPV.119577 (osteoderm).

**Locality/Horizon:** DMNH loc. 5204, “Tom’s Dirty Hole”, Lower Kirtland Formation (Campanian), San Juan County, New Mexico. Within the Ah-She-Sle-Pah Wilderness Study Area.

**Description:** DMNH EPV.119577 (Figs. 6F and 6G) is a flat, ovoid trunk osteoderm with a small, weakly-defined imbrication facet and a narrow right-lateral imbrication facet on the external surface (Fig. 6F). These two imbrication facets are connected to one another in such a way that they form a single continuous surface on the anterior and right lateral margins. The external surface of DMNH EPV.119577 is heavily ornamented with irregularly branching grooves and ridges, with subcircular pits interspersed between the ridges.

**Discussion:** Similar subrectangular/ovoid trunk osteoderms and polygonal cranial osteoderms, both with external surficial sculpting, are commonly associated with modern anguid genera, including *Ophisaurus*, *Elgaria*, *Gerrhonotus*, *Mesapsis*, *Abronia*, and *Barisia* (Mead, Arroyo-Cabrales & Johnson, 1999). Additionally, similar osteoderms attributed to Anguidae have commonly been recovered from Upper Cretaceous microvertebrate localities in Montana (Judith River Formation, Sahni, 1972), Wyoming (Lance Formation, Estes, 1964), Canada (Dinosaur Park Formation, Gao & Fox, 1996), Utah (Kaiparowits Formation, Nydam, 2013a), Texas (Aguja Formation, Nydam, Rowe & Cifelli, 2013), and Mexico (Cerro del Pueblo Formation, Aguillon-Martinez, 2010). We conditionally assign the osteoderms described herein to Anguidae due to the shape (subrectangular/ovoid for trunk osteoderms, polygonal for cranial osteoderms), and external surficial sculpting patterns (subcircular pits and narrow, irregular grooves) (Mead, Arroyo-Cabrales & Johnson, 1999). Subtle differences in an osteoderm’s external surface sculpting is commonly used for taxonomic distinction in fossil and modern anguid lizards (Meszoely & Ford, 1976; Mead, Arroyo-Cabrales & Johnson, 1999; Mead et al., 2012). We refrain from inferring lower-level taxonomic affinities until more complete specimens are recovered from the Fruitland/Kirtland formations; however, we do recognize that there at least two distinct morphotypes of osteoderm within these described specimens. “Hunter Wash” Anguidae Osteoderm Morphotype A consists of DMNH EPV.119455 and DMNH EPV.119457 (Figs. 6A–6E). The external surfaces of both osteoderms are covered in small, 50–100 µm-diameter pits that are surficial expressions of a network of vessels that radiate from roughly the center of the osteoderm. “Hunter Wash” Anguidae Osteoderm Morphotype B consists solely of DMNH EPV.119577 (Figs. 6F–6G). The external surface of “Hunter Wash” Anguidae Osteoderm Morphotype B differs from Morphotype A because it is ornamented with irregularly branching grooves and ridges, with additional subcircular pits interspersed in between the ridges.

### Systematic Paleontology

**Table:** 

Platynota Duméril & Bibron, 1836
Platynota indet.

**Referred Specimen:** DMNH EPV.119569 (jaw fragment)

**Locality and Horizon**: DMNH 5204, “Tom’s Dirty Hole”, Lower Kirtland Formation (Campanian), San Juan County, New Mexico. Within the Ah-She-Sle-Pah Wilderness Study Area.

**Figure 7:**
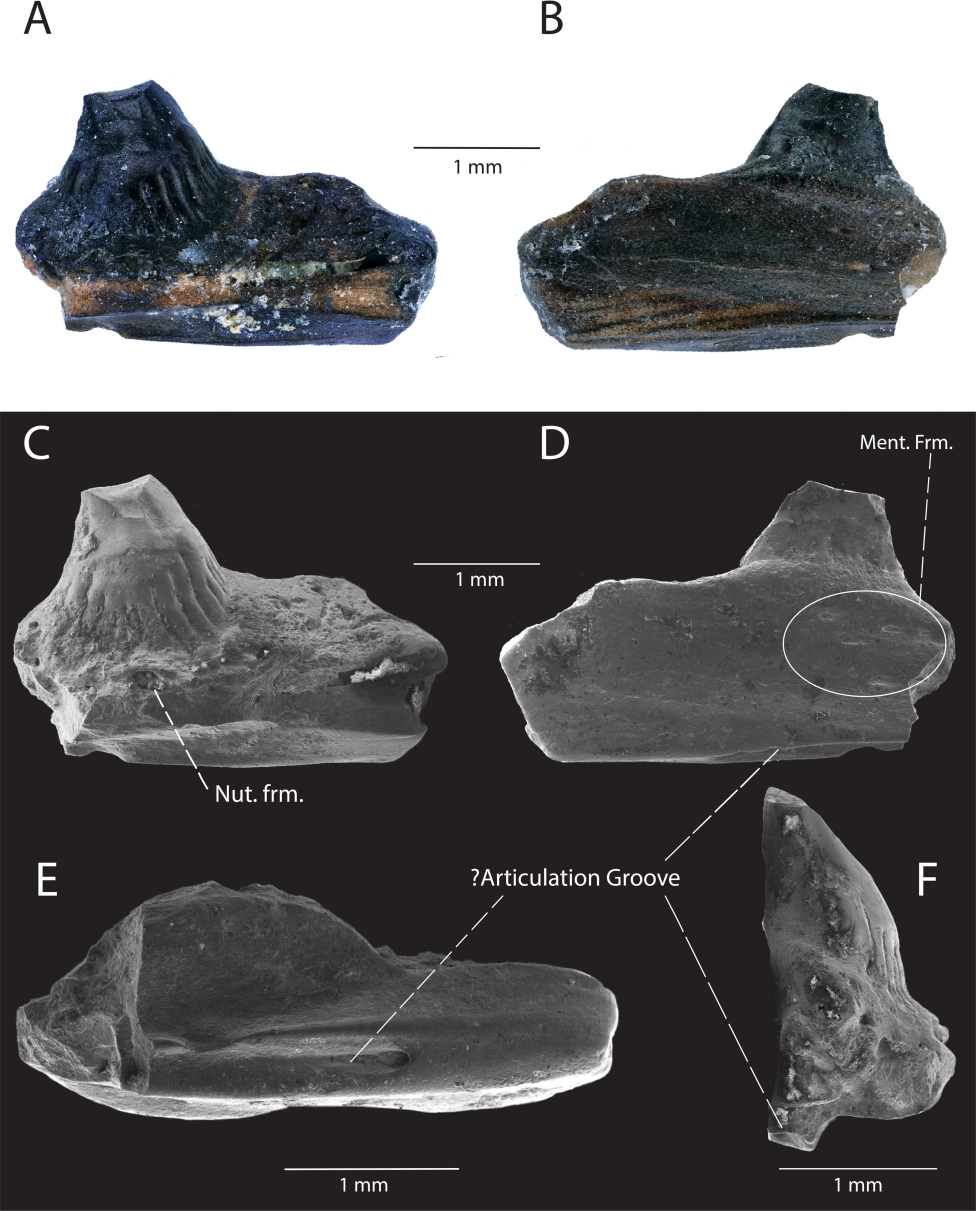
DMNH EPV.119569, Platynota indet., jaw fragment from the “Hunter Wash Local Fauna”, Kirtland Formation. (A) Medial view. (B) Lateral view. (C) Scanning electron micrograph, medial view. (D) scanning electron micrograph, lateral view. (E) Scanning electron micrograph, ventral view. (F) Scanning electron micrograph, posterior view. Abbreviations: Ment. Frm., Mental Foramina; Nut. frm, Nutrient foramina.

**Description**: DMNH EPV.119569 (Figs. 7A–7F) is a jaw fragment with two tooth positions and one partial tooth preserved. The lateral surface of the jaw is smooth with no evidence of fused osteoderms. The lateral surface swells where the preserved tooth articulates with the lateral wall of the specimen. A cluster of five posteriorly-directed mental foramina are arranged on the surface of the jaw near the articulation of the preserved tooth and the lateral parapet (Fig. 7D). On the ventrolateral/dorsolateral surface of the specimen, a v-shaped groove is directed posteriorly along the surface (Figs. 7D–7F, see ‘Discussion’ below), and terminates with an anteriorly-excavated, rounded fossa. Two tooth positions are preserved, while one tooth base with the basal portion of the crown is preserved. Tooth attachment is subpleurodont, with the lateral attachment extending one-third of the tooth height. The base of the tooth is subcircular and possesses basal infoldings that contact the circumferential ridge of cementum surrounding the base of the tooth at a sharp angle. The lingual basal infoldings terminate below the lateral parapet. A large, medially directed nutrient foramen is present at the base of the tooth (Fig. 7C). The tooth crown is broken off, but the basal portion of the crown exhibits wearing of enamel. The mesial carina (distal carina not preserved) is positioned labially.

**Discussion:** DMNH EPV.119569 is referable to Platynota on the basis of tooth morphology: **(1)** the expanded base of the tooth has been used as a diagnostic character for Platynota (Gao & Fox, 1996) and less inclusive groups within Platynota (Platynota exclusive *Dorsetisaurus pubeckensis*, Conrad, 2008); **(2)** the basal enamel infoldings have also been used to diagnose less inclusive groups within Platynota (e.g., Varanoidea, Estes, De Queiroz & Gauthier, 1988; *Parasaniwa wyomingensis* + *Parviderma inexacta* + Varanoidea, Conrad, 2008). We compared DMNH EPV.119569 to maxillae and dentaries belonging to modern platynotan specimens (*Heloderma suspectum* (LACM 165160; LACM 163853; LACM 163855), *H. horridum* (LACM 163852; LACM 163854; LACM 159136), *Varanus bengalensis* (LACM 159019; LACM 163952-54; LACM 159042), *V. salvator* (LACM 163949)), and could not find a structure similar to the posteriorly-projecting v-shaped groove. Additionally, figured specimens belonging to paracontemporaneous fossil platynotans from North America, including *Parasaniwa cynochoros* from the Kaiparowits Formation, southern Utah (UMNH VP 21180, partial right maxilla, Nydam, 2013a), c.f. *Parasaniwa wyomingensis* from the Aguja Formation, southwestern Texas (OMNH 30882, partial ?right dentary, (Nydam, Rowe & Cifelli, 2013), *Paraderma bogerti* (AMNH FARB 8504, partial right maxilla, (Sahni, 1972), and *Parasaniwa* c.f. *Parasaniwa wyomingensis* from the Cerro del Pueblo Formation, Coahuila, Mexico (SEPCP 9/582, dentary, (Aguillon-Martinez, 2010) do not showcase a v-shaped groove in a similar position. If DMNH EPV.119569 is a maxilla, the groove could be interpreted as an articulation facet with the jugal, but no structure exists in such an extreme lateral position in the posterior maxillae of the surveyed modern platynotan specimens. If DMNH EPV.119569 is a dentary, then the v-shaped groove could be interpreted as the anterior termination of the Meckelian canal, but DMNH EPV.119569 is not as curved as the symphyseal regions of the observed modern platynotan specimens, and the Meckelian canal is primarily exposed medially at the anterior end of the dentary. Alternatively, the v-shaped groove could be an articulation surface with the lateral articulation facet of the coronoid or surangular, but modern platynotans observed in the collection at NHMLA and figured fossil platynotan specimens in primary literature (see above for specimen numbers and references) do not exhibit an articulation surface so ventrally-located beneath the posterior end of the dentary. The foramen and groove could also be neurovascular in nature. The unique position of the groove on DMNH EPV.119569 may be an autapomorphic feature of this particular platynotan, however more complete material is needed to determine the anatomical relationships of this structure.

The presence of Platynota in the “‘Hunter Wash Local Fauna” of the Fruitland and Kirtland formations’ is to be expected, as the group has a wide distribution in Late Campanian deposits in Laramidia, including the Cerro del Pueblo Formation (Aguillon-Martinez, 2010) in Coahuila, Mexico, the Aguja Formation in southwestern Texas, USA (Nydam, Rowe & Cifelli, 2013), the Kaiparowits Formation in southern Utah, USA (Nydam, 2013a), the Mesaverde Formation in central Wyoming, USA (DeMar & Breithaupt, 2006), the Judith River Formation in northern Montana, USA (Sahni, 1972), and the Dinosaur Park Formation in southeastern Alberta, Canada (Gao & Fox, 1996). Given the rich history fossil vertebrate collection in the Fruitland and Kirtland formations, however, it is unexpected and significant that no platynotan material had been collected or described until the study herein. DMNH EPV.119569 offers the first definitive evidence of platynotan lizards in the Late Campanian of New Mexico, and fills in a critical gap in the regional distribution of Platynota in Laramidia.

## Discussion

### Squamate diversity and biogeography on Laramidia

The fossils described above represent important additions to the record of Late Cretaceous lizards in the San Juan Basin. The lizard-bearing fossil localities from this study in the “Hunter Wash Local Fauna” (DMNH loc. 6685, “Black Bowl”, Upper Fruitland Formation; DMNH loc. 5204, “Tom’s Dirty Hole”, Lower Kirtland Formation) preserve different taxa with no observed overlap. Because both sites occur well within the same time-averaged faunal assemblage at the gradational contact of the Upper Fruitland and Lower Kirtland formations (Bauer, 1916; Clemens, 1973; Hunt & Lucas, 1992; Sullivan & Lucas, 2003; Lucas, Hunt & Sullivan, 2006; Sullivan & Lucas, 2006), any taxonomic differences between the two sites are interpreted here to be a byproduct of local depositional environment, taphonomy, and/or incomplete sampling, rather than true faunal differences through time. The non-chamopsiid scincomorphan specimen (DMNH EPV.119554, Fig. 3) and the platynotan specimen (DMNH EPV.119569, Fig. 7) represent the first described occurrences of their respective groups within the Fruitland and Kirtland formations, while the chamopsiid specimen (DMNH EPV.119583, Fig. 2) and the anguid specimens (DMNH EPV.119555, Fig. 5; DMNH EPV.119455, Figs. 6A–6B; DMNH EPV.199457, Figs. 6C–6E; DMNH EPV.119577. Figs. 6F–6G) represent additions to previous descriptions based on fragmentary data (Armstrong-Ziegler, 1978; Armstrong-Ziegler, 1980; Sullivan, 1981). This has intriguing implications not only for the diversity of lizards within the Fruitland and Kirtland formations, but also for regional and continental biogeographic patterns and lizard family-level dispersal in Late Campanian North America.

**Figure 8:**
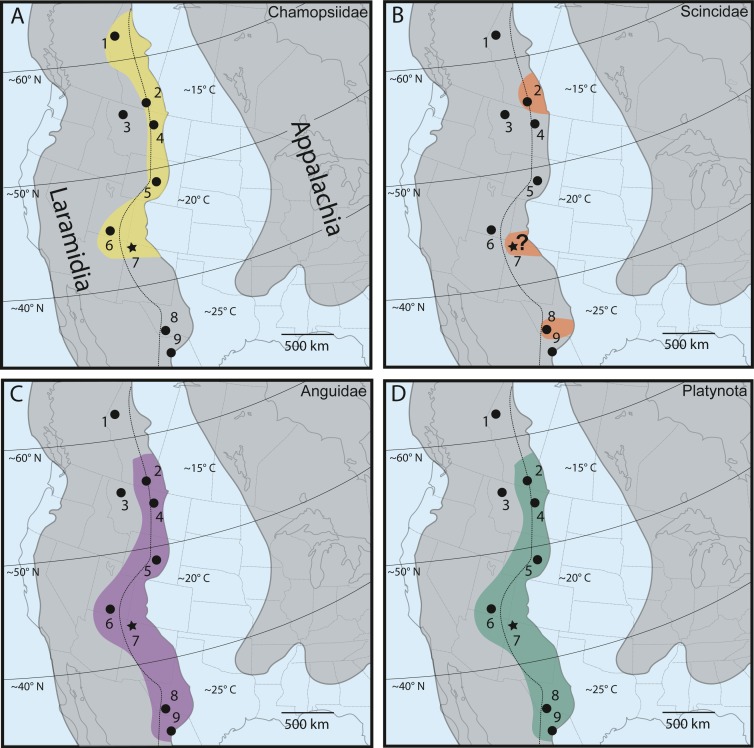
Late Campanian latitudinal distribution of lizard groups described in this study, modified from Nydam (2013b), Nydam, Rowe & Cifelli (2013) and Wiersma & Irmis (2018). Dots indicate squamate-bearing Campanian geologic units, with star representing locality of newly described Fruitland/Kirtland specimens. (A) Distribution of Chamopsiidae (yellow). (B) Distribution of Scincidae (orange). Question mark at the Fruitland/Kirtland occurrence indicates uncertainty of specimen DMNH EPV.119554 belonging to Scincidae. (C) Distribution of Anguidae (violet). (D) Distribution of Platynota (teal). Geologic Units: (1) Wapiti Formation (Nydam, Caldwell & Fanti, 2010). (2) Dinosaur Park Formation (Gao & Fox, 1996). (3) Two Medicine Formation (DeMar et al., 2012). (4) Judith River Formation (Sahni, 1972). (5) Mesa Verde Formation (DeMar & Breithaupt, 2006). (6) Kaiparowits Formation (Nydam, 2013a; Nydam, Eaton & Sankey, 2007; Nydam & Voci, 2007; Nydam & Fitzpatrick, 2009). (7) Fruitland/Kirtland formations (Armstrong-Ziegler, 1978; Armstrong-Ziegler, 1980; Sullivan, 1981; Present Study). (8) Aguja Formation (Nydam, Rowe & Cifelli, 2013). (9) Cerro del Pueblo Formation (Aguillon-Martinez, 2010). Dotted line indicates approximate boundary between the alluvial plain depositional environment (west of line) and the coastal plain depositional environment (east of line) (Gates et al., 2010). Paleolatitudes from Lehman (1997) and included references. Mean annual paleotemperature estimates from Lehman (1997) and included references, Miller et al. (2013), Estrada-Ruiz, Martínez-Cabrera & Cevallos-Ferriz (2010), Gates et al. (2010) and included references, and Manchester, Lehman & Wheeler (2010).

As discussed extensively in previous work (Nydam, 2013b; Nydam, Rowe & Cifelli, 2013), chamopsiids are commonly found throughout mid-latitude and higher-latitude localities in Laramidia, while no specimens have been reported south of the paleolatitude of 40°N (Fig. 8A). This pattern suggests that Chamopsiidae was restricted to mid-to-northern ecosystems on Laramidia and might have only tolerated subtropical climates (Nydam, Rowe & Cifelli, 2013). Although tentative chamopsiid material (discounting *Leptochamops* material since identified as amphibian) has been described from the “Hunter Wash Local Fauna” of the Fruitland and Kirtland formations (Armstrong-Ziegler, 1980; Sullivan, 1981; present study), the overall record for the unit remains extremely fragmentary. What makes chamopsiids from the Fruitland and Kirtland formations especially important is that they occupy the southernmost known distribution for the family during the Late Campanian. The Fruitland and Kirtland formations could therefore be critical for future work assessing chamopsiid distributional patterns at geographic “faunal zone” boundaries (Gates et al., 2010). This synergistic biogeographic work necessitates revision of extremely common Late Campanian chamopsiid genera such as *Chamops* and *Leptochamops*, as well as recovering more complete material in field surveys, in order to describe detailed geographic distribution and diversity patterns in this family (Gao & Fox, 1996; Nydam & Voci, 2007; Nydam, 2013b; Nydam, Rowe & Cifelli, 2013).

Although the lower-level taxonomic affinities of the scincomorphan specimen (DMNH EPV.119554, Fig. 3) cannot be determined from its morphological characteristics alone, it shares enough overlapping features with modern scincids and Late Campanian North American scincids to be included in a tentative biogeographic comparison (Fig. 8B). Scincids are comparatively rare in the Late Campanian of Laramidia, but have been reported from the Dinosaur Park Formation in Alberta (Gao & Fox, 1996) and the Aguja Formation in southwestern Texas (Nydam, Rowe & Cifelli, 2013). The “Hunter Wash” scincid-grade specimen adds an important mid-paleolatitude data point to the distribution of the clade on Laramidia, but occurrences in comparison to other lizard families in Late Campanian Laramidia remains rare (Fig. 8), either due to true lack of abundance, lack of preservation, and/or undersampling.

Late Campanian anguids, which are almost entirely comprised of the genus *Odaxosaurus*, have been reported from every paracontemporaneous geologic unit from Laramidia except for the Two Medicine Formation in Montana, USA, and the Wapiti Formation in Alberta, Canada (Nydam, Rowe & Cifelli, 2013). Anguids range from the Cerro del Pueblo Formation in Coahuila, Mexico (Aguillon-Martinez, 2010) to the Dinosaur Park Formation in Alberta, Canada (Gao & Fox, 1996) (Fig. 8C). DMNH EPV.119555, *Odaxosaurus* sp., from the “Hunter Wash Local Fauna” adds to a growing dataset of occurrences of *Odaxosaurus* in Southern Laramidia. With three named species (*O. piger*, Aguja Formation, southwestern Texas, (Nydam, Rowe & Cifelli, 2013); *O. priscus, O. roosevelti*, Kaiparowits Formation, southern Utah, (Nydam, 2013a) and one potential new species (*O.* sp. nov., Cerro del Pueblo Formation, Coahuila, Mexico, (Aguillon-Martinez, 2010), *Odaxosaurus* represents the only multi-specific lizard genus known from the Late Campanian of North America (Table 2). Though DMNH EPV.119555 is too poorly preserved to permit species-level identification, it would not be surprising if it represented a distinct species of *Odaxosaurus*, given the presence of at least three different species of *Odaxosaurus* mentioned above in geographically-bracketing lizard faunas in southern Laramidia.

The platynotan specimen is the first described occurrence of predatory lizards in the Fruitland and Kirtland formations. Platynotans were widely distributed in the Campanian of North America, with specimens reported from the Cerro del Pueblo Formation in Coahuila, Mexico (Aguillon-Martinez, 2010), to the Dinosaur Park Formation in Alberta (Gao & Fox, 1996) (Fig. 8D). Given this near ubiquitous distribution of Platynota in Laramidia, the recovery of platynotan specimens from the Fruitland/Kirtland formations is predictable. However, the fact that DMNH EPV.119569 is the only recovered Platynota specimen from the Fruitland/Kirtland formations suggests that their abundance in microvertebrate assemblages in the Late Campanian of New Mexico is relatively low, either due to a true low abundance, preservation bias, or collection bias.

## Conclusions

The new chamopsiid, scincomorphan, anguid, and platynotan material from the “Hunter Wash Local Fauna” of the Fruitland and Kirtland formations of northwestern New Mexico represent important primary data points in examining lizard diversity and distribution in both a local faunal assemblage and on a regional paleogeographic scale. The two lizard-bearing fossil localities in the “Hunter Wash Local Fauna” DMNH loc. 6685, “Black Bowl”, and DMNH loc. 5204, “Tom’s Dirty Hole”, preserve lizard taxa exclusive to one another. This suggests that local depositional environment, taphonomy, and/or incomplete sampling are major sources of bias in the preservation of specific lizard taxa within the Fruitland/Kirtland formations—a phenomenon observed in most other fossil squamate localities from Laramidia (Nydam, 2013b). On a regional scale, the squamate fauna of the Fruitland and Kirtland formations fills in critical gaps in the geographic range of widely-distributed squamate clades from Laramidia. While it is to be expected that the described taxa in this study can be found in the Fruitland and Kirtland formations, their presence alone in an understudied mid-paleolatitude microvertebrate assemblage represents a substantial addition to the already robust dataset of lizard assemblages from Laramidia.

## Institutional abbreviations

DMNH/DMNS: Denver Museum of Nature & Science, Denver, Colorado, USA
MNA: Museum of Northern Arizona, Flagstaff, Arizona, USA
NMHLA/LACM: Natural History Museum of Los Angeles County, Los Angeles, California, USA
OMNH: Sam Noble Oklahoma Museum of Natural History, Norman, Oklahoma, USA
SEPCP: Secretaria de Educacion y Cultura, Coleccion Paleontologica, Coahuila, Mexico
UALP: University of Arizona Laboratory of Paleontology, Tucson, Arizona, USA
UMNH: Natural History Museum of Utah, Salt Lake City, Utah, USA
UNM-FKK: Department of Geology, University of New Mexico, Albuquerque, New Mexico, USA

## Additional Information and Declarations

### Field Study Permissions

The following information was supplied relating to field study approvals (i.e., approving body and any reference numbers): All specimens described in this study were collected under United States Federal Bureau of Land Management permit NM14-04S.

### Data Availability

The following information was supplied regarding data availability: No coding was necessary to carry out experiments in this study. All raw data (described fossils) included in this study are provided within submitted manuscript. All specimens described in this study are housed in the Earth Sciences Vertebrate Paleontology Collections at the Denver Museum of Nature & Science (DMNH). Specimens DMNH EPV.119455 and DMNH EPV.119457 (“Hunter Wash” Anguidae Osteoderm morphotype A) are accessioned under DMNH accession number 2016-161. Specimens DMNH EPV.119554 (Scincomorpha left dentary), DMNH EPV.119555 (Anguidae left dentary), DMNH EPV.119567 (Scincomorpha osteoderm), DMNH EPV.119569 (Platynota dentary), DMNH EPV.119577 (“Hunter Wash” Anguidae Osteoderm Morphotype B), DMNH EPV.119583 (Chamopsiidae jaw fragment) are accessioned under DMNH accession number 2014-264.
